# A new scoring system for prediction of fibrosis in chronic hepatitis C

**Published:** 2011-07-01

**Authors:** Simona Bota, Roxana Sirli, Ioan Sporea, Mircea Focsa, Alina Popescu, Mirela Danila, Mihnea Strain, Madalina Sendroiu, Alexandra Deleanu, Isabel Dan

**Affiliations:** 1 Department of Gastroenterology and Hepatology, University of Medicine and Pharmacy, Timisoara, Romania; 2 Department of Biophysics and Medical Informatics, University of Medicine and Pharmacy, Timisoara, Romania

**Keywords:** liver fibrosis, chronic HCV hepatitis, Elastography, Serological test

## Abstract

**Background:**

Liver biopsy (LB) is still considered to be the gold standard for assessment of liver fibrosis.

**Objectives:**

To evaluate the effectiveness of various non-invasive methods for predicting liver fibrosis, including transient elastography (TE), APRI score, Lok score, Forns score, FIB-4 score, Fibrosis Index, King score, and Bonacini score, in comparison with the effectiveness of LB and to create a new scoring system for fibrosis prediction.

**Patients and Methods:**

This study included 212 patients with chronic HCV hepatitis. LB, TE, and various biological tests were performed during a single hospital visit. Using established formulae, data from these tests were used to create scores for assessment of liver fibrosis.

**Results:**

The results of all the tests showed significant correlation with histological fibrosis. TE results (r = 0.62), King score (r = 0.57), and APRI score (r = 0.56) showed the closest correlation with severity of fibrosis. The following formula was derived from our data by multiple regression: Predicted liver fibrosis score (PLF score) = 0.956 + 0.084 × TE - 0.004 × King score + 0.124 × Forns score + 0.202 × APRI score. A direct correlation (r = 0.68) was found between the PLF score and liver fibrosis. The cut-off values of the PLF score for various stages of fibrosis were: F ≥ 1, 1.77 (Area under ROC curve (AUROC) = 0.76); F ≥ 2, 2.18 (AUROC = 0.78); F ≥ 3, 2.47 (AUROC = 0.86); and F = 4, 2.98 (AUROC = 0.97).

**Conclusions:**

We found that our newly developed PLF score, which is derived from the scores of multiple tests, is more strongly correlated with fibrosis than each component score used individually. The PLF score is more effective than TE for predicting severe fibrosis, but they have similar effectiveness in predicting liver cirrhosis.

## 1. Background

Chronic hepatitis, caused by hepatitis C virus (HCV), is an important public health problem. In 1999, the World Health Organization estimated the global prevalence of HCV infection to be approximately 3%, with the disease affecting around 170 million people [1]. In Europe, the prevalence is estimated to be 1%, but with large variations between countries [2]. In Romania, the prevalence is estimated to be 3.23% [3]. The assessment of liver fibrosis is important for the staging and prognosis of chronic hepatitis, and liver biopsy (LB) is still considered to be the gold standard for this purpose [4][5][6]. Recently, noninvasive approaches employing ultrasound-based technology, including transient elastography (TE)-FibroScan [7][8][9], real-time elastography [10][11][12][13][14], and acoustic radiation force impulse elastography (ARFI) [15][16][17][18][19], and serological methods, most notably, FibroTest-ActiTest, have been developed for evaluation of liver fibrosis [20][21][22][23][24].

TE is performed with the Fibroscan® device (Echosens, Paris, France), which consists of a 5-MHz ultrasound transducer probe mounted on the axis of a vibrator. The vibrator generates a completely painless vibration (with a frequency of 50 Hz and amplitude of 2 mm), which generates an elastic share wave propagating through the skin and the subcutaneous tissue to the liver. The velocity of the wave is directly related to tissue stiffness [8][9]. ARFI elastography is based on the principle that compression of the examined tissue induces less strain in hard than in softer tissues. The ultrasound probe automatically produces an acoustic "push" pulse that generates shear-waves, which propagate into the tissue. Using image-based localization and proprietary ARFI technology, shear wave speed and tissue depth may be quantified in a precise anatomical region of interest predefined by the system. Propagation speed, measured in meters/second (m/s), is displayed on a screen and increases with fibrosis severity [17][18].

Other biological scores, such as APRI score, Lok score, Forns score, FIB-4 score, FI-Fibrosis Index score, King score, and Bonacini score are very simple to calculate using standard biological tests and are used in daily practice [25][26][27][28][29][30][31]. Currently, TE is the most commonly used non-invasive method for assessment of liver fibrosis, particularly in Europe. TE has not only been validated in HCV chronic hepatitis but also in HBV chronic hepatitis, non-alcoholic steatohepatitis (NASH), and post-transplant patients with primary biliary cirrhosis (PBC) [9][32][33][34].

## 2. Objectives

The aim of this study was to evaluate the effectiveness of various noninvasive methods for predicting liver fibrosis, including TE-FibroScan, APRI score, Lok score, Forns score, FIB-4 score, FI (Fibrosis Index) score, King score, and Bonacini score, in comparison with the effectiveness of current gold standard of LB. On the basis of the results, we aimed to create a new scoring system for predicting liver fibrosis with increased sensitivity, specificity, and accuracy than the individual scoring systems.

## 3. Patients and Methods

### 3.1. Patients

This was retrospective study that included 212 patients with chronic HCV hepatitis (143 women and 69 men; mean age 49.9 ± 9.9 years) admitted to our department from January 2008 to March 2010. The patients were anti-HCV positive for at least 6 months and had detectable levels of HCV-RNA by RT-PCR. All patients underwent abdominal ultrasound, LB, liver stiffness (LS) measurements by means of TE, and biological tests. Informed consent was obtained from all patients, and the study protocol was approved by the local ethics committee.

### 3.2. Liver biopsy

Echo-assisted LB was performed in all patients by using modified Menghini needles (1.4 and 1.6 mm in diameter). Only LB fragments including at least 8 portal tracts were considered adequate for pathological interpretation and were included in our study. The LBs were assessed according to the Metavir scoring system by a senior pathologist blinded to the results of the LS measurements. Fibrosis was staged on a 0-4 scale: F0, no fibrosis; F1, portal fibrosis without septa; F2, portal fibrosis and few septa extending into lobules; F3, numerous septa extending to adjacent portal tracts or terminal hepatic venules; and F4, cirrhosis.

### 3.3. Transient elastography

LS was measured by means of TE using a FibroScan device (EchoSens-Paris, France). In each patient, we performed 10 valid TE measurements, after which the median value was calculated and the results were expressed in kPa. In this study, we included only LS measurements with a success rate (the ratio of the number of successful acquisitions over the total number of acquisitions) of at least 60% and an interquartile range (IQR) lower than 30%. (IQR is the difference between the 75th and 25th percentile, essentially the range of the middle 50% of the data).

### 3.4. Serological score

Bioassays were performed by venous blood sampling and were processed in our hospital's laboratories. All bioassays were routine biological tests and the following normal values (NV) were used: aspartate aminotransferase (AST), NV = 5-34 U/L; alanine aminotransferase (ALT), NV = 10-35 U/L; gamma glutamyl transpeptidase (GGTP), NV = 12-64 U/L; platelet count, NV = 150000-450000/mm³; cholesterol, NV < 200 mg%; serum albumin, NV = 3.5-5 g/dL; and INR, NV = 0.88-1.10. On the basis of these biological tests, we calculated the following scores for predicting liver fibrosis:

APRI score = [(AST/upper limit NV AST) ×100]/number of platelets (10⁹/l) [25]

Lok score: log odds = -5.56 - 0.0089 × number of platelets (10³/mm³) + 1.26 × (AST/ALT) + 5.27 × INR

Lok = [exp (log odds)]/ [1 + exp (log odds)] [26]

Forns score = 7.811 - 3.131 × ln [number of platelets (10⁹/l)] × 0.781 ln [GGTP (U/L)] + 3.467 × ln [age (years)] - 0.014 [cholesterol (mg/dl)] [27]

FIB-4 score = [age (years)] × AST (U/L)]/[number of platelets (10⁹/L)] × ALT (U/L)(½)] [28]

FI score (fibrosis index) = 8 - 0.01 × number of platelets (10⁹/L) - albumin (g/dl) [29]

King score = age (years) × AST (U/L) × INR/number of platelets (10⁹/L) [30]

Bonacini score: different points are given (which are added together) according to the value of AST/ALT, INR and platelet count [31](Table 1).

**Table 1:** Bonacini score

**Parameter**	**0 points**	**1 point**	**2 points**	**3 points**	**4 points**	**5 points**	**6 points**
**INR**	< 1.1	1.1-1.4	> 1.4	-	-	-	-
**ALT/AST**	> 1.7	1.7-1.2	1.19-0.6	< 0.6	-	-	-
**Platelets **(x10³/mm ³)	> 340	340-280	279-220	219-160	159-100	99-40	< 40

### 3.5. Statistical analysis

Data were collected and tabulated in a Microsoft Excel file. Statistical analysis was performed using the MedCalc program and WINK Statistical Data Analysis Research Software. The LS values and the scores were expressed as means and standard deviation. Spearman's rank correlation coefficient was used to assess correlations between histological findings and the various predictive scores of liver fibrosis. Two-way ANOVA was used to compare mean values for different stages of fibrosis in the new scoring system. Multiple regression was used to calculate the new liver fibrosis scores. The diagnostic performance of the new scoring system was assessed using receiver operating characteristic (ROC) built for the detection of fibrosis: (F ≥ 1, Metavir score), significant fibrosis (F ≥ 2), severe fibrosis (F ≥ 3), and cirrhosis (F = 4). Optimal cut-off values were chosen so that sensitivity and specificity were maximal. Sensitivity and specificity were calculated according to standard methods. Further, 95% confidence intervals were calculated for each predictive test and were used to compare AUROC curves.

## 4. Results

According to the Metavir scoring system, the severity of liver fibrosis in the study group of 212 patients with chronic hepatitis C was graded as follows: 1.4% (3 patients) had no fibrosis (F = 0); 8.0% (17 patients) had stage 1 fibrosis (F = 1); 44.8% (95 patients) had stage 2 fibrosis (F = 2); 31.6% (67 patients) had stage 3 fibrosis (F = 3); and 14.2% (30 patients) had cirrhosis (F = 4). The average fragment size obtained by LB was 3.35 ± 0.90 cm. All methods used for predicting liver fibrosis were directly, and significantly, correlated with histological findings, but TE (r = 0.62), King score (r = 0.57), and APRI score (r = 0.56) had the strongest correlation with fibrosis severity (Table 2). We chose the 4 tests that had the strongest correlation to severity of fibrosis in histological samples, and from these data, we used multiple regression to develop a new score (PLF score) for predicting the severity of liver fibrosis. The formula employed in this analysis was as follows:

PLF score = 0.956 + 0.084 × TE - 0.004 × King score + 0.124 × Forns score + 0.202 × APRI score.

**Table 2:** Correlation between different tests and liver fibrosis (assessed according Metavir score)

**Test**	**Spearman's rank correlation coefficient **	**P value**
**TE-FibroScan**	0.62	< 0.0001
**King score**	0.57	< 0.0001
**APRI score**	0.56	< 0.0001
**Forns score**	0.55	< 0.0001
**Lok score**	0.49	< 0.0001
**FI a score**	0.49	< 0.0001
**FIB-4 score**	0.45	< 0.0001
**Bonacini score**	0.42	< 0.0001

^a^ FI: Fibrosis index

A direct correlation (Spearman co-efficient; r = 0.68) was found to exist between our new scoring system and the Metavir scoring system (P < 0.0001). The correlation of the new scoring system with the severity of fibrosis was better than that of the other methods alone. The mean PLF scores for different stages of fibrosis ranged from 1.93 ± 0.45 for F0 to 3.64 ± 0.55 for F4 (Table 3). While there was no significant difference between mean PLF scores for F0 and F1 stages of fibrosis (P = 0.77), statistically significant differences were apparent for F1 vs. F2 (P = 0.01), F2 vs. F3 (P < 0.001), and F3 vs. F4 (P < 0.001). Using the ROC curve, we calculated the cut-off PLF scores for different stages of liver fibrosis (Table 4).

The PLF score had a better predictive value than the TE score for significant fibrosis (F ≥ 2: AUROC = 0.78 vs. 0.74 [P = 0.02]), and for severe fibrosis (F ≥ 3: AUROC = 0.86 vs. 0.81 [P = 0.003]). However, for cirrhosis, the predictive values were similar (AUROC = 0.97 vs. 0.97; P = 0.28) (Table 5). The PLF score also had a better predictive value than the King score for severe fibrosis (F ≥ 3: AUROC = 0.86 vs. 0.81 [P = 0.02]) and for cirrhosis (F = 4: AUROC = 0.97 vs. 0.88 [P = 0.001]). However, the 2 tests had similar effectiveness in predicting significant fibrosis (F ≥ 2: AUROC = 0.78 vs. 0.75 [P = 0.07]) (Table 5). The value of the PLF score was significantly higher than the Forns score for predicting significant fibrosis (F ≥ 2: AUROC = 0.78 vs. 0.73 [P = 0.01]), severe fibrosis (F ≥ 3: AUROC = 0.86 vs. 0.80 [P = 0.006]) and cirrhosis (F = 4: AUROC = 0.97 vs. 0.85 [P = 0.0001]) (Table 5). The PLF score was also better than the APRI score for predicting significant fibrosis (F ≥ 2: AUROC = 0.78 vs. 0.68 [P = 0.003]) and cirrhosis (F = 4: AUROC = 0.97 vs. 0.87 [P = 0.0006]), while the 2 tests were similarly effective in predicting severe fibrosis (F ≥ 3: AUROC = 0.86 vs.0.82 [P = 0.058]) (Table 5).

**Table 3:** The mean values of PLF score for different stages of fibrosis (according to Metavir score)

**Fibrosis grade**	**Patients, **No.	**Prediction of liver fibrosis score, **Mean ± SD
**F = 0**	3	1.93 ± 0.45
**F = 1**	17	1.99 ± 0.32
**F = 2**	95	2.18 ± 0.27
**F = 3**	67	2.57 ± 0.41
**F = 4**	30	3.64 ± 0.55

**Table 4:** Cut-off values of PLF score for different stages of fibrosis (according to the Metavir score system)

**fibrosis Stage**	**Cut-off value**	**AUROC**^a^	**Se**^b^**, **%	**Sp**^c^**, **%	**PPV**^d^**, **%	**NPV**^e^**, **%	**Accuracy,** %
**F ≥ 1**	1.77	0.76	95.6	66.6	99.5	18.1	95.2
**F ≥ 2**	2.18	0.78	71.3	75	96.4	21.4	71.6
**F ≥ 3**	2.47	0.86	71.1	89.5	85.1	78.6	81.1
**F = 4**	2.98	0.97	96.6	93.4	70.7	99.4	93.8

^a^ AUROC: Area under ROC curve

^b^ Se: Sensitivity

^c^ Sp: Specificity

^d^ PPV: Positive predictive value

^e^ NPV: Negative predictive value

**Table 5:** Comparation between AUROC curves for PLF scor and TE, King score, Forns score , APRI score for prediction of different stages of fibrosis (according to the Metavir score system)

	**Fibrosis grade**	**Difference between area**	**Standard error**	**95% CI **d	***P* value**
**AUROC**^a^** PLF **b**score *vs.* AUROC TE**^c^					
**0.789 *vs.* 0.742**	F ≥ 2	0.0472	0.0298	0.0275 to 0.0828	0.02
**0.862 *vs.* 0.810**	F ≥ 3	0.0524	0.0177	0.0177 to 0.0871	0.003
**0.972 *vs. *0.977**	F = 4	0.00441	0.00412	-0.00367 to 0.0125	0.28
**AUROC PLF score *vs.* AUROC King score**					
**0.789 *vs.* 0.759**	F ≥ 2	0.030	0.0392	-0.0165 to 0.103	0.07
**0.862 *vs.* 0.815**	F ≥ 3	0.0477	0.0218	0.0058 to 0.0904	0.02
**0.972 *vs.* 0.887**	F = 4	0.0847	0.0262	0.0331 to 0.136	0.001
**AUROC PLF score *vs.* AUROC Forns score**					
**0.789 *vs.* 0.735**	F ≥ 2	0.054	0.0314	0.0294 to 0.0936	0.01
**0.862 *vs.* 0.804**	F ≥ 3	0.0581	0.0215	0.1159 to 0.100	0.006
**0.972 *vs.* 0.852**	F = 4	0.120	0.0305	0.607 to 0.180	0.0001
**AUROC PLF score *vs.* AUROC APRI score**					
**0.789 *vs.* 0.688**	F ≥ 2	0.101	0.0377	0.0531 to 0.153	0.003
**0.862* vs.* 0.825**	F ≥ 3	0.037	0.0243	-0.00156 to 0.0935	0.058
**0.972 *vs.* 0.879**	F = 4	0.0932	0.0272	0.0398 to 0.147	0.0006

^a^ AUROC: Area under curve

^b^ PLF: Predicted liver fibrosis score

^c^ TE: Transient elastography

^d^ CI: Confidence interval

## 5. Discussion

While noninvasive methods are being increasingly used for the assessment of liver fibrosis, clinicians must choose between different serological tests [35] and elastographic methods. The APRI scoring system is not expensive and is accessible to all clinicians. A meta-analysis [25] conducted in 2007 showed that with a cut-off value of 0.5, APRI scores had 81% sensitivity (Se) and 50% specificity (Sp) for predicting significant fibrosis (F ≥ 2, Metavir score), and with a cut-off value of 1, they had 76% Se and 71% Sp for predicting cirrhosis. Using these cut-off values in the current study, we obtained 67.7% Se, 70% Sp, 95.5% positive predictive value (PPV), 70% negative predictive value (NPV), and 67.9% accuracy for predicting significant fibrosis. For prediction of cirrhosis, we obtained 80% Se, 74.1% Sp, 33.8% PPV, 95.7% NPV, and 75% accuracy. The Lok score was originally proposed by Lok and co-workers [26]. According to the authors, a cut-off value of < 0.2 excludes the diagnosis of liver cirrhosis (7.8% of patients wrongly classified) and a value greater than 0.5 predicts the diagnosis of liver cirrhosis (14.8% of patients wrongly classified). In our study, only 1 patient with liver cirrhosis had a Lok score < 0.2 (3.3% of the patients with cirrhosis were incorrectly classified) and 4 patients without cirrhosis had a Lok score > 0.5 (2.1% of patients without cirrhosis were incorrectly classified).

According to published studies, the accuracy of the Forns score for predicting significant fibrosis ranges from 50% to 83% [27][36]. This scoring system was initially used in HIV-HCV co-infected patients and was subsequently validated in HCV patients. With a cut-off value of 6.9 for the presence of significant fibrosis, 96% of patients were correctly classified [37]. In our study group, only 59.2% of patients were classified correctly using this cut-off value. The FIB-4 score was also originally developed for HIV-HCV co-infected patients but has subsequently proved effective for predicting severe fibrosis in HCV patients (F3 and F4) with an AUROC of 0.85. Using a cut-off value of < 1.45, the FIB-4 score excludes the presence of severe fibrosis with a NPV of 94.7% (74% Se and 80% Sp) and with a cut-off value greater than 3.25, it can confirm the presence of severe fibrosis with a PPV of 82% (37.6% Se and 78% Sp) [28][38]. In the current study, using the same cut-off value for prediction of severe fibrosis, we obtained 21.6% Se, 99.1% Sp, 95.4% PPV, 60% NPV, and 63.6% accuracy. In a previous study, using a cut-off value of < 2.10, the FI score had 66.8% Se and 78.8% PPV for predicting F0-F1 fibrosis, and 68.5% sensitivity and 68.6% PPV in the validation group [29]. In addition, with a cut-off value of ≥ 3.30, FI had 67.7% Se and 75% PPV for predicting cirrhosis, while the sensitivity was 70.8% and PPV was 81% in the validation group [29]. In the current study, using these cut-off values for prediction of F0-F1 fibrosis, we obtained 95% Se, 21.8% Sp, 11.2% PPV, 97.6% NPV, and 61% accuracy. For predicting liver cirrhosis, FI had 13.3% Se, 99.4% Sp, 50% PPV, 84.1% NPV, and 85.8% accuracy. In a previous study, the King score predicted cirrhosis with 86% Se, 80% Sp, and 96% NPV using a cut-off value ≥ 16.7 [30]. In the current study, we obtained 90% Se, 74.1% Sp, 36.4% PPV, 97.8% NPV, and 76.4% accuracy. The King score was also found to be the serological test that had the strongest correlation with fibrosis (Spearman coefficient, r = 0.57).

The Bonacini formula uses the Bonacini score (as calculated above) in combination with an evaluation of the liver surface by abdominal ultrasound to predict severe fibrosis and cirrhosis [31]. An algorithm based on these data was used to predict cirrhosis and correctly classified 67% of patients as having high (> 75%) or low (< 10%) risk of cirrhosis, with only 33% of the patients requiring LB to confirm the diagnosis [31]. TE is a method that has been proven to be useful for predicting significant fibrosis (F ≥ 2), with cut-off values ranging from 7.1 to 8.7 kPa, and cirrhosis (F = 4) with cut-off values ranging from 12.5 to 14.5 kPa [39][40]. In a meta-analysis published by Friedrich-Rust et al., using a cut-off value of 7.65 kPa, TE had an excellent predictive value for the diagnosis of cirrhosis (AUROC = 0.94) and a good predictive value for significant fibrosis (AUROC = 0.85) [32]. In a study by Sporea et al. [33], the optimal cut-off value for predicting significant fibrosis was 6.8 kPa, with 56.5% Se, 94.7% Sp, 97.5% PPV, and 37.5% NPV, while the optimal cut-off value for predicting severe fibrosis (F ≥ 3) was 10.1 kPa [34]. In another study published by Sirli et al. [41], using a cut-off value of 13.3 kPa, TE had 93.3% Se, 96.1% Sp, 73.7% PPV, and 99.2% NPV (AUROC = 0.97) for predicting cirrhosis. In the current study, using a cut-off value of 6.8 kPa for the presence of significant fibrosis, TE had 61.4% Se, 85% Sp, 97.5% PPV, 18.6% NPV, and 63.6% accuracy. Using a cut-off value of 10.1 kPa for the presence of severe fibrosis, TE had 52.5% Se, 93.9% Sp, 87.9% PPV, 93.9% NPV, and 75% accuracy. With a cut-off value of 13.3 kPa, TE had 93.3% Se, 97.2% Sp, 84.8% PPV, 98.8% NPV, and 96.6% accuracy for predicting cirrhosis.

In a study published by Sirli et al. [41], various non-invasive methods for evaluation of liver fibrosis were compared to LB. An inverse correlation with fibrosis was obtained for platelet count (r = -0.484, P < 0.0001), and direct correlations were obtained for the APRI score (r = 0.570, P < 0.0001), TE-FibroScan (r = 0.569, P < 0.0001), Forns score (r = 0.540, P < 0.0001), Lok score (r = 0.484, P < 0.0001), and FIB-4 score (r = 0.417, P < 0.0001). In the current study, the methods that correlated most strongly with fibrosis were TE-FibroScan (r = 0.62, P < 0.0001), King score (r = 0.57, P < 0.0001), APRI score (r = 0.56, P < 0.0001), and Forns score (r = 0.55, P < 0.0001). In a study performed by Friedrich-Rust [15], in which ARFI was compared to LB and blood markers in 86 patients with chronic hepatitis (HBV or HCV), the Spearman correlation co-efficients between histologically determined fibrosis and ARFI, TE, FibroTest, and APRI scores, were 0.71, 0.73, 0.66, and 0.45 respectively, and these values were statistically significant (P < 0.001).

In a study published in 2005 by Castera et al. [39], 183 patients with chronic HCV hepatitis were evaluated by LB, TE, Fibrotest, and APRI. The AUROC curves for FibroScan, FibroTest, and APRI in prediction of significant fibrosis (F ≥ 2), severe fibrosis (F ≥ 3), and cirrhosis (F = 4) were respectively 0.83, 0.85, and 0.78; 0.90, 0.90, and 0.84; and 0.95, 0.87, and 0.83. The most effective prediction performance was obtained by combining the FibroScan and FibroTest scores with AUROC curves of 0.88 for F ≥ 2, 0.95 for F ≥ 3, and 0.95 for F = 4. When the FibroScan and FibroTest results agreed, LB confirmed the diagnosis in 84% of cases for F ≥ 2, 95% for F ≥ 3, and 94% for F = 4. In another study published in 2010, Castera et al. [21] studied 2 algorithms for prediction of liver fibrosis: one utilized TE and FibroTest and the other used APRI and FibroTest (SAFE biopsy). LB was also performed in all patients. Significant fibrosis (F ≥ 2) was present in 76% of patients and cirrhosis (F4) in 25%. TE failure was observed in 8 cases (2.6%). For significant fibrosis, the Castera algorithm prevented the need for 23% more liver biopsies (71.9% vs. 48.3%, respectively, P < 0.0001) than did SAFE biopsy, but its accuracy was significantly lower (87.7% vs. 97.0%, respectively; P < 0.0001). While the accuracy of the Castera algorithm in predicting cirrhosis was significantly higher than that of SAFE biopsy (95.7% vs. 88.7%, respectively; P < 0.0001), the number of liver biopsies required did not differ between the 2 algorithms (78.8% vs. 74.8%; P = NS).

Shahenn published a meta-analysis in which he compared the performances of TE and Fibrotest (in patients with chronic HCV hepatitis) for prediction of liver fibrosis [42]. Data were collected from 13 studies, 9 for FibroTest (1679 patients) and 4 for TE (546 patients). In heterogeneous analyses for significant fibrosis, the AUROC curves for FibroTest and TE were 0.81 (95% CI, 0.78-84) and 0.83 (0.03-1.00), respectively. At a threshold of approximately 0.60, the sensitivity and specificity of the FibroTest was 47% (35-59%) and 90% (87-92%), respectively. For TE (at a threshold of approximately 8 kPa), the corresponding values were 64% (50-76%) and 87% (80-91%). However, the diagnostic accuracy of both measures was correlated with the prevalence of significant fibrosis and cirrhosis in the study populations. For cirrhosis, the summary AUROC curves for FibroTest and FibroScan were 0.90 (95% CI, not calculable) and 0.95 (0.87-0.99), respectively.

In a study published in 2010 by Cross et al. [43], 187 patients with chronic HCV hepatitis were evaluated on the basis of LB, TE, and King score. Liver fibrosis was scored using the Ishak score, with significant fibrosis being defined as an Ishak score of F3-F6 and cirrhosis being defined as an Ishak score of F5-F6. The AUROC curves for TE, King score, and TE + King score for the diagnosis of Ishak F3-F6 were 0.83, 0.82, and 0.85, respectively, and those for the diagnosis of cirrhosis (F ≥ 5, Ishak score) were 0.96, 0.89, and 0.93, respectively. The NPVs for diagnosis of cirrhosis using the optimal cut-off values for TE (10.05 kPa), King score (24.3), and both combined (26.1) were 98%, 91%, and 94%, respectively. In a study published by Wang et al. [44], 214 patients with chronic HCV hepatitis, 88 patients with chronic HBV hepatitis and 18 patients with chronic HBV + HCV hepatitis were evaluated by LB, TE, and ultrasonography (US). US scores, including those obtained after assessment of liver surface, liver parenchyma, intrahepatic vessels, and spleen index, were used to assess the degree of hepatic fibrosis. LS measurements as determined by TE correlated significantly with hepatic fibrosis scores, necro-inflammatory activity, and US scores in multivariate analysis. The diagnostic accuracy of TE in the prediction of all HCV-related fibrosis scores was significantly superior to that of US and was equal to that of TE and US combined. In a study published by Sporea et al. [45], 242 subjects (171 with LB and 71 with clinical, ultrasonographic, endoscopic, and/or laparoscopic signs of cirrhosis) were evaluated by TE and ARFI. A direct correlation was found between TE measurements and fibrosis (r = 0.858), between ARFI and fibrosis (r = 0.784), and also between TE and ARFI (r = 0.740). The optimal cut-off value for prediction of significant fibrosis (F ≥ 2) was 7.1 kPa for TE (AUROC = 0.92, 80% Se, 95% Sp) and 1.2 m/s for ARFI (AUROC = 0.90, 85% Se, 88% Sp) and that for prediction of cirrhosis (F = 4) was 13.8kPa for TE (AUROC = 0.98, 95% Se, 94% Sp) and 1.8 m/s for ARFI (AUROC = 0.92, 91% Se, 87% Sp). When both values of TE and ARFI were higher than the cut-off values, they achieved 65% Se and 98% Sp for prediction of significant fibrosis (F ≥ 2) and 74% Se and 97% Sp for prediction of cirrhosis. In the cases in which one of the values was higher than the cut-off value, they achieved 90% Se and 84% Sp for prediction of significant fibrosis and 98% Se and 84% Sp for prediction of cirrhosis.

In this study, there was no significant difference between the mean values of the PLF score for F0 and F1 stages of fibrosis (P = 0.77), which may have been due to the small number of patients included in these 2 groups (3 patients with F0 and 1 with F1 in the LB group). However, the differences were statistically significant for F1 vs. F2 (P = 0.01), F2 vs. F3 (P < 0.001), and F3 vs. F4 (P < 0.001). The PLF score had a better predictive value than did TE for significant fibrosis (F ≥ 2: AUROC = 0.78 vs. 0.74 [P = 0.002]) and for severe fibrosis (F ≥ 3: AUROC = 0.86 vs. 0.81 [P = 0.003]), while the predictive values for cirrhosis were similar: AUROC = 0.97 vs. 0.97 (P = 0.28). The PLF score also had better predictive values for different stages of fibrosis than did the King score (with the exception of F ≥ 2, Metavir score), Forns score, and APRI score (with the exception of F ≥ 3, Metavir score). In future studies, we will validate the PLF score in other groups of patients. For prediction of F ≥ 1 using a cut-off value of 1.77, the PLF score had 95.6% Se, 99.5% PPV, and 95.2% accuracy. For prediction of significant fibrosis (F ≥ 2), with a cut-off value of 2.18, the PLF score had a 96.4% PPV, while for prediction of fibrosis (F = 4), with a cut-off value of 2.98, the PLF score had 96.6% Se, 93.4% Sp, 99.4% NPV, and 93.8% accuracy.

In conclusion, we have devised a new PLF scoring system, derived from TE and multiple serological tests, to predict the severity of liver fibrosis. PLF scores are more closely correlated with fibrosis than each of the individual tests when used alone (r = 0.68). While the new scoring system is more effective than TE (FibroScan) in predicting significant and severe fibrosis, their predictive values for cirrhosis are similar.
